# Trimethylamine N-Oxide (TMAO) Inducing Endothelial Injury: UPLC-MS/MS-Based Quantification and the Activation of Cathepsin B-Mediated NLRP3 Inflammasome

**DOI:** 10.3390/molecules28093817

**Published:** 2023-04-29

**Authors:** Dongyu Lei, Wenbo Yu, Yi Liu, Yujie Jiang, Xiaohui Li, Jing Lv, Ying Li

**Affiliations:** 1 Department of Pharmacology, Xiangya School of Pharmaceutical Sciences, Central South University, Changsha 410078, China; 2 Department of Physiology, School of Basic Medicine, Xinjiang Medical University, Urumqi 830017, China; 3 Department of Health Management, The Third Xiangya Hospital, Central South University, Changsha 410013, China

**Keywords:** Trimethylamine-N-Oxide, UPLC-MS/MS, endothelialinjury, NLRP3 inflammasome, Cathepsin B

## Abstract

TMAO is a new risk biomarker for cardiovascular disease. With trimethylammonium as its main chemical skeleton, TMAO is structurally similar to many endogenous metabolites, such as acetylcholine, carnitine, phosphorylcholine, etc. The mechanism of TMAO on the pathological process of CVD is still unclear. In this study, the quantitative analysis of plasma TMAO is conducted, and the contribution of Cathepsin B and NLRP3 inflammasome during the process of TMAO-induced endothelial injury was proposed and investigated at animal and cellular levels. Immunofluorescence assay was applied to represent the protein expression of Cathepsin B and NLRP3 inflammasome located at endothelial cells. The results showed that TMAO could disrupt endothelial cells permeability to induce endothelial injury, meanwhile, TMAO could increase NLRP3 inflammasome activation and promote the activity and expression of Cathepsin B in vitro and in vivo, whereas inhibition of NLRP3 inflammasome activation by MCC950 could protect the endothelial cells from TMAO associated endothelial injury via Cathepsin B. The study reveals that TMAO can cause endothelial injury via Cathepsin B-dependent NLRP3 inflammasome, and inhibition of Cathepsin B and NLRP3 inflammasome can reduce the TMAO-induced damage. The results provide new insight into the role of TMAO in CVD, which can be a potential therapeutic target for disease treatment and drug design.

## 1. Introduction

Trimethylamine N-oxide (TMAO), as a metabolite from dietary choline such as L-carnitine, etc., has been thought to be a novel risk biomarker for cardiovascular disease (CVD) [1,2,3,4]. TMAO is oxidized by hepatic flavin-dependent monooxygenases (FMOs) from trimethylamine (TMA) metabolized by intestinal microbiota absorbed and transported to the liver [5]. It has been proven to be closely related to many diseases such as hypertension, atherosclerosis, heart failure and vascular aging [6,7,8]. TMAO embraces trimethylammonium as its main chemical skeleton, which is the building block of many endogenous metabolites such as acetylcholine, phosphorylcholine, phosphatidylcholine, etc. The structural similarities of functional groups between TMAO and endogenous metabolites might interfere with the metabolism and result in cardiovascular disorders. However, the mechanism of TMAO on the pathological process is still unclear, which restricts drug design and disease control.

Endothelial injury plays an important role in the pathological process of cardiovascular diseases and it is commonly characterized by enhanced endothelium permeability, which is typically controlled by the tight junction proteins, such as ZO-2, OCLN, etc. [9,10,11]. Endothelial nitric oxide synthase (eNOS) also contributes to the regulation of endothelial function and acts as a master regulator of vascular tone and homeostasis [12]. The endothelial injury can result in and be accelerated by the inflammatory responses of endothelial cells [13]. NLR-family pyrin domain-containing protein 3 (NLRP3), as an important inflammasome, has a close connection with multiple chronic inflammatory diseases and metabolic disorders such as obesity, hypertension, diabetes, and so on [14,15]. It has been found that an increased TMAO level can induce the activation of NF-kappa B (NF-κB) pathway and increase the expressions of inflammatory factors, including TNF-α and IL-1β [16]. Additionally, Cathepsin B (CTSB) is also concerned within the regulation of inflammation and plays an essential role in pathological conditions. It is reported that NLRP3-dependent endothelial cell pyroptosis could be activated by HMGB1/RAGE/Cathepsin B signaling [17]. Therefore, it is proposed that the increment of TMAO stimulation might result in endothelial injury by NLRP3 inflammasome activation.

In the present work, the plasma concentration of TMAO is investigated based on UPLC-MS/MS, and the relationships between TMAO and the levels of endothelial injury and inflammation related factors, such as eNOS, Cathepsin B, and NLRP3 are studied at the animal and cellular levels. This study aims to understand the critical role of TMAO in vascular endothelial injury and provide guidance for the structure-based drug design and disease control on CVD.

## 2. Results and Discussion

### 2.1. Quantification of Plasma TMAO Based on UPLC-MS/MS and Its Effects on Endothelial Injury

**Figure 1:**
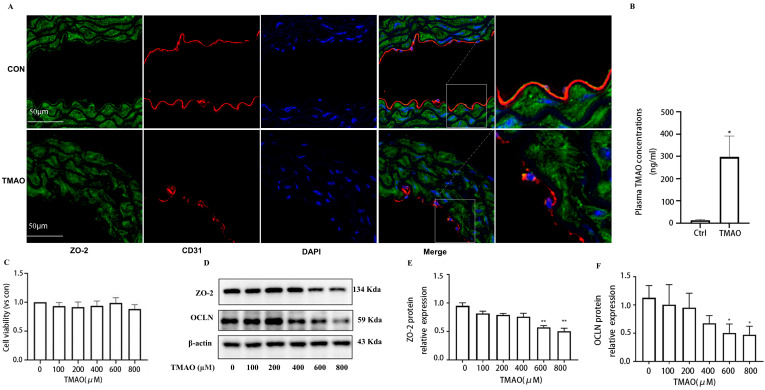
Effects of TMAO on endothelial cell junction in vivo and in vitro. (**A**) Location of ZO-2 (green) in the endothelial cells of thoracic aorta was estimated by immunofluorescence microscopy, the nucleus was labeled with DAPI (blue) and endothelial marker CD31 was labeled with red. (**B**) Mice serum TMAO concentration was determined by UPLC-MS/MS. (**C**) Cell viability was determined by CCK8. (**D**) Western blot analysis of ZO-2 and OCLN in HUVECs treated with TMAO for 24 h in different concentrations. (**E**,**F**) Bar charts show the quantification of the indicated proteins. *, Compared with the control group, * *p* < 0.05, ** *p* < 0.01. *n* = 3.

**Figure 2:**
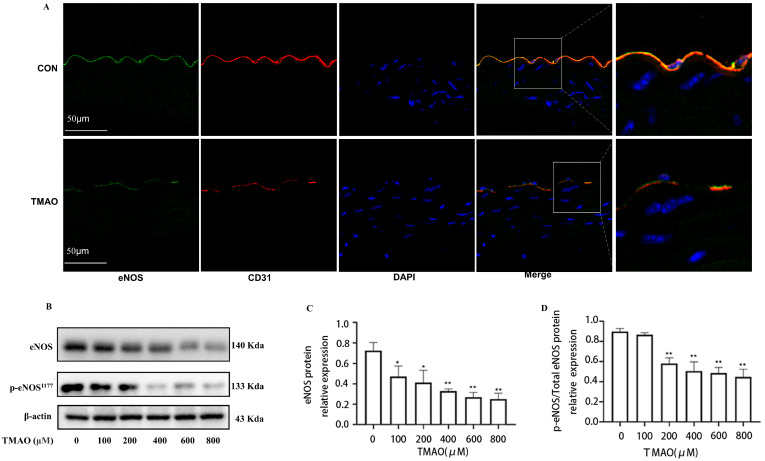
Effects of TMAO on endothelial function in vivo and in vitro. (**A**) Location of eNOS (green) in the endothelial cells of thoracic aorta was estimated by immunofluorescence microscopy, the nucleus was labeled with DAPI (blue) and Endothelial marker CD31 was labeled with red. (**B**) Western blot analysis of p-eNOS/eNOS in HUVECs treated with TMAO for 24 h in different concentrations. (**C**,**D**) Bar charts show the quantification of the indicated proteins. *, Compared with the control group, * *p* < 0.05, ** *p* < 0.01. *n* = 3.

To investigate the relationships between TMAO and factors contributing to endothelial injury, a high-TMAO-drink-induced mice model and the quantitative analysis method of plasma TMAO based on UPLC-MS/MS were established. Since TMAO always exists in plasma with its precursors, such as Choline, Carnitine, Betaine, etc., the contents of the above compounds in plasma samples are detected, and the high sensitivity and selectivity method was established. The results showed that the plasma TMAO concentration of the TMAO-drinking mice (300 ng/mL) is significantly increased compared with the control mice (Figure 1B). With the increment of plasma TMAO, the expression of ZO-2 and eNOS were decreased in the endothelial cells of thoracic aorta of TMAO drinking mice by immunofluorescence co-location assay (Figure 1A and Figure 2A). In addition, cell viability determination showed that after the treatment of HUVECs with various concentrations of TMAO for 24 h, TMAO exerted no significant effects on cell viability (Figure 1C), but induced a significant decrease in ZO-2, OCLN (Figure 1D–F) and p-eNOS/eNOS expression (Figure 2B–D) in HUVECs exposed to TMAO for 24 h in a dose-dependent manner. Endothelial junction proteins ZO-2 and OCLN have been reported to regulate cells’ permeability, therefore, downregulation of these proteins could lead to junctional disruption and enhanced cellular permeability. Endothelial inflammation plays an important role in initiating the occurrence and progression of endothelial injury [18]. The results indicated that TMAO triggered endothelial injury in vivo and in vitro.

### 2.2. TMAO Upregulates the Expression of Cathepsin B In Vivo and In Vitro Accompanied by Increased Activity of Cathepsin B

**Figure 3:**
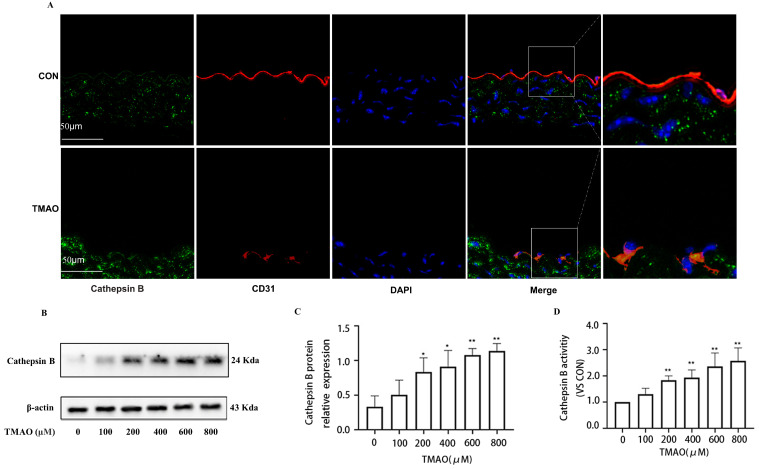
Effects of TMAO on the expression and activity of Cathepsin B in vivo and in vitro. (**A**) Location of Cathepsin B (green) in the endothelial cells of thoracic aorta was estimated by immunofluorescence microscopy, the nucleus was labeled with DAPI (blue) and Endothelial marker CD31 was labeled with red. (**B**) Cells were treated with TMAO for 24 h with different concentrations, the expression of Cathepsin B was analyzed by western blot. (**C**) Bar charts show the quantification of the indicated proteins. (**D**) Cells were treated with TMAO at the indicated concentrations, the Cathepsin B activity in the HUVECs was detected by Cathepsin B activity assay. *, Compared with the control group, * *p* < 0.05, ** *p* < 0.01. *n* = 3.

To investigate the relationship between Cathepsin B and TMAO, the level of Cathepsin B is detected in the endothelial cells of thoracic aorta of TMAO drinking mice by immunofluorescence co-location assay. It was found that the expression of Cathepsin B of treated mice was increased compared with that of normal-drinking mice in the thoracic aorta endothelial cells (Figure 3A). Furthermore, the expression of Cathepsin B was increased in HUVECs exposed to different concentrations of TMAO (Figure 3B,C), which was accompanied with increased activity of Cathepsin B (Figure 3D). These results suggest that the expression and activity of Cathepsin B were induced by TMAO. The results revealed that TMAO could activate Cathepsin B and further prompt inflammation in endothelium.

### 2.3. TMAO Causes Endothelial Injury via Cathepsin B

**Figure 4:**
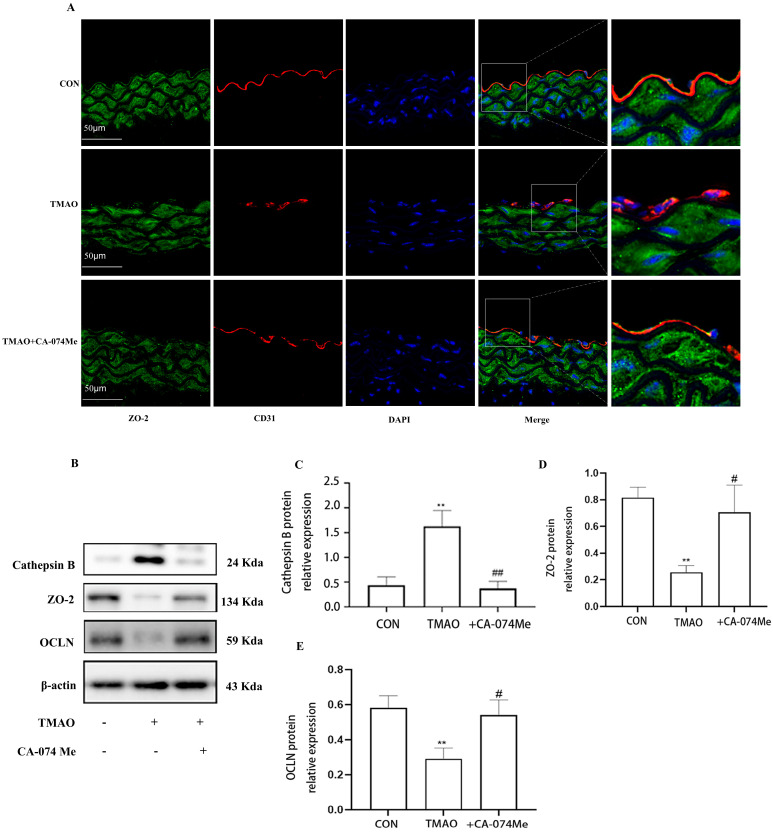
TMAO causes endothelial cell disjunction via Cathepsin B in vivo and in vitro. (**A**) Location of ZO−2 (green) in the endothelial cells of thoracic aorta was estimated by immunofluorescence microscopy, the nucleus was labeled with DAPI (blue), and endothelial marker CD31 was labeled with red. (**B**) Cells were pretreated with CA-074Me and then exposed to TMAO (600 μmol/L) for 24 h. Expression of Cathepsin B, ZO-2 and OCLN was detected via Western blot. (**C**–**E**)) Bar charts show the quantification of the indicated proteins. *, Compared with the control group, ** *p* < 0.01. #, Compared with the TMAO group, # *p* < 0.05. ## *p* < 0.01. *n* = 3.

**Figure 5:**
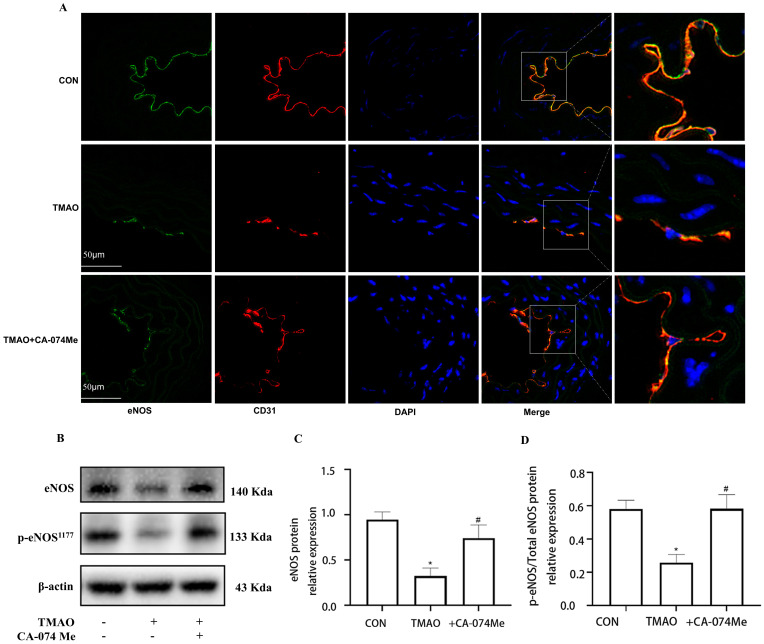
TMAO disrupts endothelial function through Cathepsin B in vivo and in vitro. (**A**) Location of eNOS (green) in the endothelial cells of thoracic aorta was estimated by immunofluorescence microscopy, the nucleus was labeled with DAPI (blue), and Endothelial marker CD31 was labeled with red. (**B**) Cells were pretreated with CA−074Me and then exposed to TMAO (600 μmol/L) for 24 h. The levels of p-eNOS/eNOS was detected via Western blot. (**C**,**D**) Bar charts show the quantification of the indicated proteins. *, Compared with the control group, * *p* < 0.05. #, Compared with the TMAO group, # *p* < 0.05. *n* = 3.

To verify the involvement of Cathepsin B in TMAO-induced endothelial injury, intraperitoneal injection Cathepsin B inhibitor CA-074Me was administered to the high-TMAO-drink-induced mice model. As shown in Figure 4A, CA-074Me could repair the TMAO-induced decreased endothelial junction protein ZO-2 in the endothelial cells of thoracic aorta in mice model. Meanwhile, CA-074Me was used before TMAO (600 μM) incubation to reveal the role of Cathepsin B in TMAO-induced endothelial injury in HUVECs. It was found that CA-074Me could also repair the TMAO-induced decreased inter-endothelial junction protein ZO-2 and OCLN in HUVECS (Figure 4B–D). Furthermore, the down-regulated expression level of eNOS were recovered by CA-074Me in the high-TMAO-drink-induced mice model (Figure 5A). Finally, CA-074Me can repair the TMAO-induced decreased expression and phosphorylation level of eNOS in HUVECs (Figure 5B–D). These results suggest that Cathepsin B is critical for a TMAO induced endothelial injury.

### 2.4. TMAO Causes Endothelial Injury via Cathepsin B/NLRP3 Inflammasome Axis

**Figure 6:**
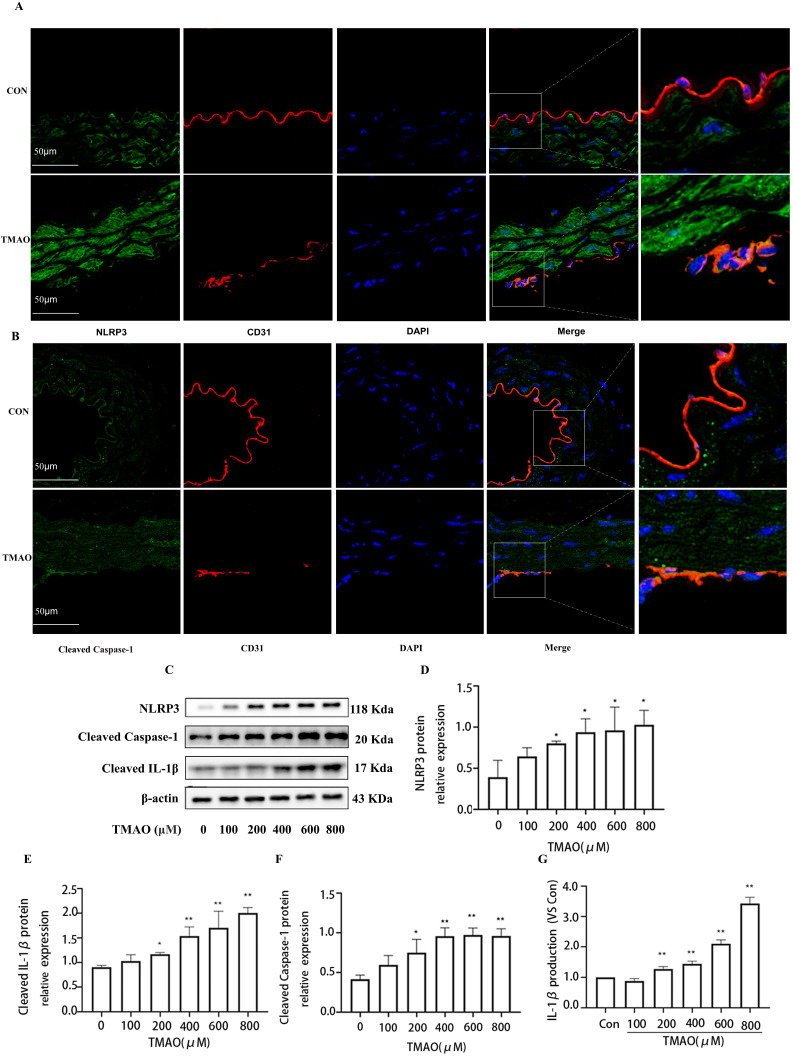
TMAO promotes NLRP3 inflammasome activation in vivo and in vitro. (**A**,**B**) Location of NLRP3 and Cleaved Caspase-1 in endothelial cell of TMAO drinking mice was estimated by immunofluorescence microscopy, the nucleus was labeled with DAPI (blue) and endothelial marker CD31 was labeled with red. (**C**) Cells were treated with TMAO at different concentrations for 24 h, and the expression of NLRP3, Cleaved Caspase-1 and Cleaved IL-1β detected via Western blot. (**D**–**F**) Bar charts show the quantification of the indicated proteins. (**G**) supernatant IL-1B levels were detected by Elisa assay. *, Compared with the control group, * *p* < 0.05, ** *p* < 0.01. *n* = 3.

To verify the involvement of Cathepsin B in induction of NLRP3 inflammasome activation in TMAO condition, firstly, the activation of NLRP3 inflammasome under TMAO administration was investigated in vivo and in vitro. As shown in Figure 6A,B, the levels of NLRP3 and Cleaved Caspase-1 were increased in the endothelial cells of thoracic aorta of TMAO-drinking-mice by immunofluorescence co-location assay. In addition, TMAO promoted NLRP3, Cleaved Caspase-1 and Cleaved IL-1β protein expression in a concentration dependent manner in HUVECS (Figure 6C–F). Furthermore, it was found that IL-1B levels of cell supernatant were upregulated compared with control group (Figure 6G). The collective results suggested that TMAO treatment promoted NLRP3 inflammasome activation in vivo and in vitro and resulted in the release of the cell supernatant IL-1B inflammatory cytokine.

**Figure 7:**
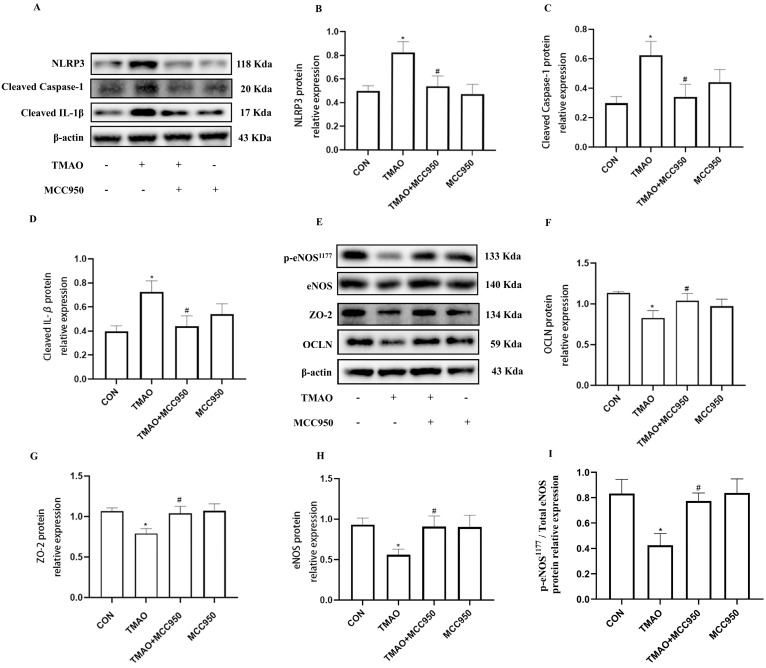
Effects of NLRP3 inhibition by MCC950 on TMAO−induced endothelial injury. (**A**) Cells were pretreated with MCC950 (10 μmol/L) for 2 h, and then exposed to TMAO (600 μmol/L) for a further 24 h. Expression of NLRP3, Cleaved Caspase−1 and Cleaved IL−1β were detected via Western blot. (**B**–**D**) Bar charts show the quantification of the indicated proteins. (**E**) Cells were pretreated with MCC950 and then exposed to TMAO (600 μmol/L) for 24 h. Expression of ZO−2, OCLN and p−eNOS/eNOS were detected via Western blot. (**F**–**I**) Bar charts show the quantification of the indicated proteins. *, Compared with the control group, * *p* < 0.05. #, Compared with the TMAO group, # *p* < 0.05. *n* = 3.

**Figure 8:**
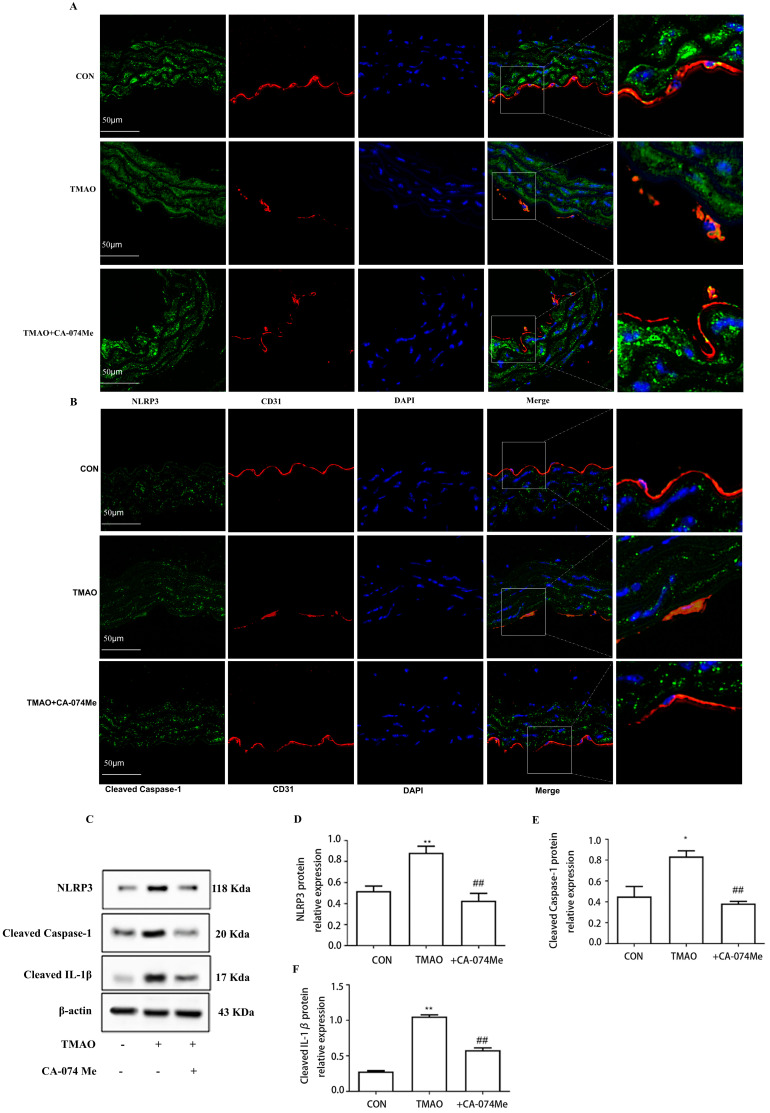
Effects of Cathepsin B inhibition by CA−074Me on TMAO−induced activation of NLRP3 inflammasome in vivo and in vitro. (**A**,**B**) Location of NLRP3 and Cleaved Caspase−1 (green) in the endothelial cells of thoracic aorta was estimated by immunofluorescence microscopy, the nucleus was labeled with DAPI (blue), and Endothelial marker CD31 was labeled with red. (**C**) Cells were pretreated with CA−074Me (10 μmol/L) for 2 h, and then exposed to TMAO (600 μmol/L) for a further 24 h, expression of NLRP3, Cleaved Caspase−1 and Cleaved IL−1β was detected via Western blot. (**D**–**F**) Bar charts show the quantification of the indicated proteins. *, Compared with the control group, * *p* < 0.05. ** *p* < 0.01. #, Compared with the TMAO group, ## *p* < 0.01. *n* = 3.

To further demonstrate the role of NLRP3 inflammasome in TMAO-induced endothelial injury, NLRP3 inhibitor MCC950 was used. By pretreating HUVESs with MCC950 (10 μmol/L) prior to the treatment with TMAO (600 μmol/L) for 24 h, it was found that MCC950 not only markedly inhibited NLRP3, Cleaved Caspase-1, and Cleaved IL-1β protein expression (Figure 7A–D), but also inhibited endothelial injury followed by increased inter-endothelial junction ZO-2 and OCLN protein expression, and the levels of p-eNOS/eNOS compared with TMAO group (Figure 7E–I). Together, these results suggest that NLRP3 inflammasome is involved in TMAO-induced endothelial injury. Finally, to verify the involvement of Cathepsin B in the induction of NLRP3 inflammasome activation in TMAO conditions, intraperitoneal injection Cathepsin B inhibitor CA-074Me was administered to the high-TMAO-drink-induced mice model. As shown in Figure 8A,B, CA-074Me can repair the TMAO-induced increased NLRP3 and Cleaved Caspase-1 levels in the endothelial cells of thoracic aorta in mice model. Meanwhile, CA-074Me was used before TMAO (600 μM) incubation to reveal the role of Cathepsin B in TMAO-induced endothelial injury in HUVECs. As shown in Figure 8C–F, the up-regulated protein levels of NLRP3, Cleaved Caspase-1 and Cleaved IL-1β were significantly reduced by CA-074Me. The lysosomal damage and the leakage of Cathepsin B from lysosomes have been implicated as upstream events of the NLRP3 inflammasome activation stimulated by many danger factors including free fatty acids, cholesterol crystals, and *Lactobacillus casei* wall components [19,20,21]. The results suggested that Cathepsin B is critical for the TMAO induced endothelial injury via NLRP3 inflammasome activation.

## 3. Materials and Methods

### 3.1. Reagents and Materials

TMAO was purchased from Sigma, inhibitors included CA-074Me (Selleck, S7420) and MCC950 (MCE, HY-12815A). Immunoblotting was performed using primary antibodies Cathepsin B (CST, #31718), NLRP3 (CST, #15101), Cleaved IL-1β (Abcam, ab9722), Cleaved Caspase-1 (CST, #2225), eNOS (CST, #32027), p-eNOS (CST, #9570), ZO-2 (BOSTER, BA 2861), OCLN (BOSTER, BM 4832).

### 3.2. Animals and Experimental Protocols

The procedures of study were approved by the Institute of Animal Care and Ethical Committee of Hunan Normal University. During the study, the animals were fed with maintain fodder and placed under a constant temperature condition with a 12 h light/dark cycle. Male C57BL/6 mice (6 weeks old) weighing between 18 and 20 g were divided into three groups: Control, TMAO and TMAO + CA-074Me. The control group was fed with purified drinking water, while TMAO and TMAO + CA-074Me groups were fed with purified drinking water containing 0.12% TMAO after 1 week of adjustable feeding. Mice in the TMAO + CA-074Me group were injected intraperitoneally (i.p.) with a single dose CA-074Me (10 mg/kg, dissolved in Normal Saline) for 2 weeks before being sacrificed. After ten weeks, pentobarbital sodium was injected for both anesthesia (50 mg/kg body weight) and euthanasia (200 mg/kg body weight). After that, thoracic aorta was separated and immersed in paraformaldehyde immediately. Blood samples were obtained from the mice eyes, kept static placing for 2 h and centrifuge. All plasma samples and thoracic artery were stored at −80 °C for follow steps

### 3.3. Ultra Performance Liquid Chromatography-Tandem Mass Spectrometry Quantification of TMAO

Ultra Performance Liquid Chromatography-Tandem Mass Spectrometry (UPLC-MS/MS) was applied to test the plasma levels of TMAO. Plasma was extracted from mice eyes. Internal standards were added to plasma samples, vortexed (1 min) and centrifuged (12,000× *g*, 5 min, 4 °C). 2 μL of plasma sample were injected and analyzed with a column (2.1 × 50 mm, 1.7 µm) at 40 °C. The mobile phase consisted of 30% of 10 mM ammonium acetate in water (solution A) and 70% acetonitrile (solution B). The flow rate was 0.3 mL·min^−1^ [22].

### 3.4. Cell Culture and Treatments

HUVECs were received from the Pharmacy Department of The Third Hospital of Central South University and cultured in DMEM/F12 medium (Procell) supplemented with 10% fetal bovine serum (Gibco), 100 µg/mL streptomycin and 100 U/mL penicillin in a 5% CO^2^/95% air incubator at 37 °C.

### 3.5. Cathepsin B Activity Assay

The activities of Cathepsin B in HUVECs were detected via Cathepsin B activity assay kit. According to the instructions, cells were washed in cold PBS for 3 times and resuspended in 50 µL of Buffer. After being incubated in 4 °C for 30 min, the extract was centrifuged (10,000× *g*, 5 min), and 50 µL supernatant was mixed with 50 µL buffer and 2 µL substrate (10 mM) in a 96-well microplate. Plates were incubated in the dark (37 °C, 1 h) and measured on a fluorescent microplate reader (Ex/Em = 400/505 nm).

### 3.6. ELISA Assay

Culture supernatants from serious concentrations of TMAO treated HUVECs were used to analyze the secreted IL-1β by the ELISA kit (R&D Systems) according to the manufacturer’s instructions.

### 3.7. Immunofluorescence Detection

Thoracic aortas were separated from mice and fixed overnight in periodate-lysine-paraformaldehyde fixative. The fixed aortas were stained with anti-CD31 (1:100, Abcam, Cambridge, UK), anti-ZO-2 (1:200,Boster Biological Technology, Pleasanton CA, USA ), anti-OCLN (1:200, Boster Biological Technology, Pleasanton CA, USA), anti-eNOS (1:800, Abcam, Cambridge, UK), anti-p-eNOS (1:100, AdipoGen, San Diego, CA, USA), anti-Cathepsin B (1:100, AdipoGen, San Diego, CA, USA), anti-NLRP3 (1:100, AdipoGen, San Diego, CA, USA), anti-Cleaved Caspase-1 (1:100, AdipoGen, San Diego, CA, USA), anti-Cleaved IL-1β (1:100, AdipoGen, San Diego, CA, USA). Green light (Excitation Wavelength, EX): 488 nm; Red light (EX): 594 nm, Blue light (EX): 340 nm. 

### 3.8. Endothelial Cell Viability Test

The cell counting kit (CCK-8, MCE) assay was used to investigate cell viability. HUVECs (1.0 × 10^4^ cells/well) were seeded in 96 well plates with a series of concentrations of TMAO for 24 h. Then10 μL CCK-8 solutions were added into each well and incubated at 37 °C (2 h). The absorbance was measured by a microplate reader (450 nm).

### 3.9. Protein Extraction and Western Blotting

Cells were lysed on ice for 15 min in RIPA supplemented with a protease inhibitor cocktail (Biotool), and the solution was centrifugated (12,000× *g*, 15 min). The protein concentration was tested with BCA Protein Assay Kit. Proteins were then separated by sodium dodecyl sulfate polyacrylamide gel electrophoresis (SDS-PAGE) and transferred to a poly vinylidene fluoride (PVDF) membrane, which were incubated with the respective antibodies in 5% BSA at 4 °C (15 h), then it was incubated with a secondary antibody at room temperature for 2 h. The protein signals were then detected with ECL method.

### 3.10. Statistical Analyses

SPSS (software version 22.0, IBM, Chicago, IL, USA) and GraphPad Prism 8.0.2 (263) were applied to analyze the data. Quantitative data were expressed as the mean ± SD, and statistical comparisons between the groups were performed using a one-way ANOVA. A *p* value of less than 0.05 was considered as significantly different statistically.

## 4. Discussion

Plasma TMAO was first identified as a risk biomarker of CVD in 2011 [23], followed by a series of studies showing a strong association between TMAO levels and progression of CVD, including hypertension [24], atherosclerosis and cardiac hypertrophy [25], and so on. Endothelial cells contribute to forming the surface of blood vessels, the integrity of endothelium and diastolic function, which is critical for vascular homeostasis, and other functions. During the pathological process of CVD, endothelial injury has been considered to be early pathophysiological feature of CVD and an independent predictor of future cardiovascular disease [26]. A recent study reported that TMAO is associated with endothelial injury in mice and healthy humans [8]. Tight junction protein ZO-1 expression decreased remarkably with the combined action of TMAO and high glucose compared with either treatment alone in human retinal microvascular endothelial cells [27]. Our results showed that TMAO decreased inter-endothelial tight junction protein ZO-2 and OCLN expression and decreased the levels of p-eNOS/eNOS, resulting in vascular endothelial hyper permeability and reduced diastolic function, which leading to endothelial injury. These results confirmed an important role of TMAO in endothelial function which may contribute to its role in CVD as a new risk factor.

Endothelial inflammation has been known to play an essential role in endothelial injury occurrence and progression [18]. Inflammation targeted therapy embraces great potential in reducing residual cardiovascular risk. Interleukin-1 β (IL-1β) is an important pro-inflammatory cytokine in the inflammatory response of endothelial cells [28]. Although some researchers have pointed out that TMAO stimulation does not promote the expression of IL-1β mRNA levels in human aortic endothelial cells [29], it has been well documented that ingestion of choline or trimethylamine oxidase significantly increases IL-1β, leading to cellular inflammation, cardiac dysfunction, and physiological abnormalities such as arteriosclerosis [30,31]. Some reports found that the level of IL-1β could be increased when NLRP3 inflammasome was activated [32,33]. Additionally, NLRP3 inflammasome activation is related to multiple metabolic disorders and chronic inflammatory diseases, including hypertension, diabetes, atherosclerosis and so on [14,15], these diseases are closely related to the dysregulation of the endothelium. Some studies found that TMAO induces pyroptosis of vascular endothelial cells through the ALDH2/ROS/NLRP3/GSDMD signaling pathway [34]. Therefore, it can be concluded that NLRP3 inflammasome in endothelial cells is responsible for various diseases through aggravating endothelial dysfunction under pathophysiological conditions. In this study we found that TMAO enhanced the activation of NLRP3 inflammasome and caused the release of IL-1β both in vivo and in vitro, leading to the disruption of inter-endothelial junctions. Furthermore, NLRP3 inhibitor MCC950 could attenuate TMAO-induced activation of the NLRP3 inflammasome, subsequently leading to the suppression of endothelial injury in HUVECs. In a word, TMAO may accelerate endothelial inflammatory injury via activating NLRP3 inflammasome and IL-1β release. Continuously elevated TMAO levels may lead to a chronic inflammatory response in the cardiovascular system.

This study also revealed a possible mechanism underlying inflammation induced by TMAO in which Cathepsin B plays a critical role during this process. In recent years, some reports propose that multiple models can activate NLRP3 inflammasome [35,36], among which the lysosome rupture model is considered to be a major mechanism [37]. The model showed the activation of NLRP3 inflammasome by particle activators such as alum and silicon. Subsequently, lysosomal protein Cathepsin B is released into the cytoplasm, which directly or indirectly triggers the activation of inflammatory through an uncharacterized pathway. It has been reported that the inhibition of Cathepsin B in the presence of particulate activators can reduce NLRP3 inflammasome activation in human cells [38]. In this study, we first observed that the expression and active of Cathepsin B was elevated in human umbilical vein endothelial cells (HUVECs) and increased Cathepsin B was also observed in the endothelial cells of thoracic aorta under TMAO treatment. Meanwhile, Cathepsin B inhibitor CA-074Me reduced the expression of Cathepsin B in the endothelial cells of thoracic aorta under TMAO treatment. Furthermore, CA-074Me inhibited NLRP3 inflammasome activation and endothelial injury under TMAO conditions in vitro and in vivo. Our results revealed that TMAO can activate cathepsin B and further prompt inflammation in endothelium.

Many efforts have been made to determine the direct target of TMAO. The trace amine-associated receptor 5 is reported to be activated by TMA [39]; some reports suggest that TMAO may alter signaling and promote inflammatory responses by G-protein-coupled receptors located on the cell surface, such as the related molecule TMA [40]. Others pointed out that TMAO acts as a chemical chaperone [41]. In addition, some studies have also shown that TMAO is a small molecule with hydrophobicity and hydrophilicity, which can enter cells. For example, TMAO can promote metabolic dysfunction by binding and activating pancreatic ER kinase (PKR)-like ER kinase (PERK) [42]. It is of great possibility that TMAO entered into the cytoplasm and disrupted lysosome stability, leading to the release of Cathepsin B which further activate the NLRP3 inflammasome. Our results demonstrated that Cathepsin B is one of the important mediators of TMAO-induced endothelial injury, Cathepsin B may be a therapeutic target for treatment or prevention of endothelial injury and associated cardiovascular diseases. Indeed, more experiments are needed to finally answer the question of whether Cathepsin B is a direct binding target of TMAO.

## 5. Conclusions

In this study, UPLC-MS/MS-based quantitative analysis method of plasma TMAO is established and new insight into the role of TMAO in cardiovascular disease is provided. This new risk factor causes endothelial injury via Cathepsin B and NLRP3 inflammasome activation, while inhibition of the Cathepsin B and NLRP3 inflammasomes significantly reduce TMAO-induced damage. A new therapeutical strategy targeting TMAO in cardiovascular disease will be considered on lysosome and inflammation inhibition.

## Data Availability

Not applicable.

## Sample Availability

Samples of the compounds are not available from the authors.
